# Multiscale Modeling of Nanoparticle Precipitation in Oxide Dispersion-Strengthened Steels Produced by Laser Powder Bed Fusion

**DOI:** 10.3390/ma17225661

**Published:** 2024-11-20

**Authors:** Zhengming Wang, Seongun Yang, Stephanie B. Lawson, Cheng-Hsiao Tsai, V. Vinay K. Doddapaneni, Marc Albert, Benjamin Sutton, Chih-Hung Chang, Somayeh Pasebani, Donghua Xu

**Affiliations:** 1 Materials Science Program, Oregon State University, Corvallis, OR 97331, USA; wangz6@oregonstate.edu (Z.W.);; 2 School of Mechanical, Industrial and Manufacturing Engineering, Oregon State University, Corvallis, OR 97331, USA; yangseo@oregonstate.edu (S.Y.); somayeh.pasebani@oregonstate.edu (S.P.); 3 Advanced Technology and Manufacturing Institute (ATAMI), Corvallis, OR 97330, USA; 4 School of Chemical, Biological and Environmental Engineering, Oregon State University, Corvallis, OR 97331, USA; 5 Electric Power Research Institute (EPRI), Charlotte, NC 28262, USA

**Keywords:** laser powder bed fusion, additive manufacturing, oxide dispersion-strengthened alloys, particle size distribution, thermodynamics, kinetics

## Abstract

Laser Powder Bed Fusion (LPBF) enables the efficient production of near-net-shape oxide dispersion-strengthened (ODS) alloys, which possess superior mechanical properties due to oxide nanoparticles (e.g., yttrium oxide, Y-O, and yttrium-titanium oxide, Y-Ti-O) embedded in the alloy matrix. To better understand the precipitation mechanisms of the oxide nanoparticles and predict their size distribution under LPBF conditions, we developed an innovative physics-based multiscale modeling strategy that incorporates multiple computational approaches. These include a finite volume method model (Flow3D) to analyze the temperature field and cooling rate of the melt pool during the LPBF process, a density functional theory model to calculate the binding energy of Y-O particles and the temperature-dependent diffusivities of Y and O in molten 316L stainless steel (SS), and a cluster dynamics model to evaluate the kinetic evolution and size distribution of Y-O nanoparticles in as-fabricated 316L SS ODS alloys. The model-predicted particle sizes exhibit good agreement with experimental measurements across various LPBF process parameters, i.e., laser power (110–220 W) and scanning speed (150–900 mm/s), demonstrating the reliability and predictive power of the modeling approach. The multiscale approach can be used to guide the future design of experimental process parameters to control oxide nanoparticle characteristics in LPBF-manufactured ODS alloys. Additionally, our approach introduces a novel strategy for understanding and modeling the thermodynamics and kinetics of precipitation in high-temperature systems, particularly molten alloys.

## 1. Introduction

The rapidly growing need for energy around the globe is calling for new advanced structural materials in order to build the next generation energy systems (e.g., nuclear fission and fusion reactors), which are expected to operate within harsh environments (e.g., high temperature and radiation). Among other materials, oxide dispersion-strengthened (ODS) alloys are emerging as promising candidates due to their well-known mechanical properties (e.g., tensile strength, corrosion resistance, creep resistance) in harsh environments derived from extensively dispersed second phase oxide nanoparticles [1,2,3,4,5,6,7,8,9,10,11,12,13,14]. Most ODS alloys contain a high-density nanometer-scale oxide particles, such as Y_2_O_3_ and Y_2_Ti_2_O_7_, which are typically introduced by solid state powder metallurgy involving multiple steps such as ball milling, hot isostatic pressing, hot rolling, and extended heat treatments [15,16,17]. This conventional manufacturing route for ODS alloys is rather time- and energy-consuming due to the limited atomic diffusivity in the solid state. Once formed, the oxide nanoparticles dispersed inside ODS alloys impede dislocation motion, giving rise to excellent high-temperature strength and creep resistance [1,2,3,4]. Additionally, nano-scale defects formed in the aforementioned processes act as sinks for helium and provide resistance to radiation damage [18,19,20,21].

The conventional techniques of fabricating ODS alloys offer limited geometrical complexity and require post-process heat treatment to manufacture serviceable parts [6,7,8,9,10,22,23,24]. In contrast, some recent studies show that laser-melting-based additive manufacturing (AM) technologies, such as laser powder bed fusion (LPBF) and laser direct energy deposition (LDED), can directly print net-shape ODS components with lightly mixed matrix powder (or wire) and oxide particles [1,2,3,4,5,24]. These AM technologies reduce labor, energy, and cost significantly because mixed powder is prepared in a light ball milling process, and oxide particles are dissolved into the melt pool and precipitated out as nano-sized oxide particles during almost instantaneous melting and cooling processes [1,2,3,4,5,25,26,27].

The mechanical properties of ODS alloys are critically determined by the characteristics (e.g., number density, size, and size distribution) of the oxide dispersoids. The AM methods, especially LPBF, offer a cooling rate several orders of magnitude higher than the conventional methods, which can potentially prevent the coarsening of oxide nanoparticles [22,23,24,28,29,30,31] and result in small particles at a high number density, as preferred for enhancing the mechanical properties. The characteristics of oxide particles are in practice controlled by the alloy composition and printing parameters, such as laser power (P) and scanning speed (V) [3,22,23,32,33,34,35]. Previous studies tested the effects of these parameters on the PSD (particle size distribution) in experiments, which required significant amounts of work using scanning electron microscopy (SEM) and transmission electron microscopy (TEM) to measure the PSD of the oxide nanoparticles [2,3,7,9,10,11,12,13,14,15]. The time-consuming printing and characterization limited the scope of exploration of the correlations between the PSD and the processing parameters. In light of this finding, a comprehensive thermokinetic model based on the fundamental physics (diffusion and clustering) of precipitation would help accelerate the optimization of the processing parameters and development of the additively manufactured ODS alloys. Moreover, a physics-based modeling framework would also reveal the detailed kinetics and mechanisms of oxide nanoparticle precipitation and evolution during an AM process.

Several models have been established to investigate the kinetic evolution of oxide nanoparticles in ODS alloys [4,8,21,22,23,36,37,38,39]. Barnard et al. developed a model framework for the thermodynamics and kinetics of yttrium–titanium oxide nucleation, growth, and coarsening in nanostructured ferritic alloys during annealing [36]. Their model is based on some available thermodynamics and kinetic data as well as density functional theory (DFT) calculations [4,21,36,37,38]. Their model showed that the composition of nano oxide particles is controlled by both the Ti/(Y + Ti) ratio and oxygen partial pressure [36]. Hin et al. and Nellis et al. built a kinetic Monte Carlo (kMC) model based on DFT calculations and experimental data as well to study the precipitation of Y-O and Y-Ti-O in α iron during isothermal and anisothermal heat treatment [8,40]. However, these models are all focused on the precipitation of oxide particles in a solid matrix (α iron). They cannot describe the evolution of the oxide in the melt pool during the cooling phase of AM. Furthermore, Wassermann et al. measured the melt pool surface temperature in the experiment and integrated it with a thermodynamics and kinetics model to examine the limits of the PSD of Y_2_O_3_ nanoparticles in LPBF [22]. Nevertheless, they assumed that all oxide particles experienced the same temperature profile that was taken along the centerline of the melt pool surface. This assumption was made because of limitations imposed by the experimental measurement and computational cost, which restricted the power of the model in capturing the breadth of the PSD of oxide particles as determined by the variations in the cooling history in the different parts of the melt pool. In addition, the temperature profiles were still measured from the experiments, which required dedicated experimental efforts. Eo et al. coupled the Kampmann and Wagner numerical model with the temperature profile calculated by finite volume method (FVM) and thermodynamic databases to elucidate the oxide particle evolution in LPBF and LDED [23]. However, their FVM model only included the substrate and laser source, without the deposited material (wire or powder bed). Also, they only selected one representative cooling curve under each condition for precipitation modeling, similar to the work of Wassermann et al. [22]. All the model frameworks referenced above either assumed Y_2_O_3_ as the only existing oxide phase (strict stoichiometry) or divided oxide particles into a discrete number of size classes. Moreover, they mostly used the thermodynamic data from the solid state.

In this study, we developed a multiscale computational framework to model the precipitation of Y-O particles in molten 316L stainless steel (316L SS) during the LPBF process and predict the PSD of oxide nanoparticles in as-fabricated 316 SS ODS alloys. This work is focused on the thermodynamics and kinetics of Y-O nanoparticle precipitation at an elevated temperature, specifically in the liquid state. This model could be used to predict the PSD of Y-O nanoparticles under different process conditions and thus propose experimental strategies toward achieving a desired PSD. The model was validated by comparison with bulk samples of the 316L SS-Y_2_O_3_ ODS alloy that were fabricated by LPBF with different laser power and scanning speed. Section 2 of this paper presents the details of the experimental preparation and the modeling framework. Section 3 reports the thermodynamic data calculated specifically for molten 316L SS, and the model-predicted PSD of Y-O nanoparticles in comparison with the experimental measurements. Finally, the factors that may affect the PSD of Y-O nanoparticles in the ODS alloy are discussed in Section 4. The strategies that could optimize the PSD in the experiment are also proposed.

The novelty of this work lies in the development of a purely modeling framework to predict the particle size distribution (PSD) of oxide nanoparticles, which offers the potential for significant reductions in experimental costs. Unlike other experimental and computational approaches that provide only a single temperature profile for the melt pool [20,21], this model offers more comprehensive temperature profiles, leading to more accurate PSD predictions. The framework captures the precipitation of oxide nanoparticles at elevated temperatures (in the liquid state) through thermodynamic and kinetic data calculations. Additionally, it can serve as a computational guide for designing experiments to achieve the desired particle size.

## 2. Methods

### 2.1. Experimental

#### 2.1.1. Feedstock Powder

The powder feedstock in this study was gas-atomized 316L austenitic SS powder (<45 µm) from Carpenter Technology (Philadelphia, PA, USA) with the chemical composition given in Table 1 and yttria powder (<1 µm) from H.C. Stack Solutions Americas (Lewiston, ME, USA). In order to prepare the precursor of 316L ODS alloys, the 316L powder was mixed with 0.8 wt.% of Yttria (Y_2_O_3_) in a planetary ball mill for 4 h using the ball to powder ratio of 3:1. Then, the mixed powder was loaded to the LPBF machine for fabricating the 316L ODS material. ODS alloys typically contain a small amount (0.2–1 wt.%) of oxide particles. The 0.8 wt.% of yttria concentration was used here because of its potential to provide good particle detectability while preventing the significant coarsening of oxide nanoparticles [1,2,3].

**Figure 1:**
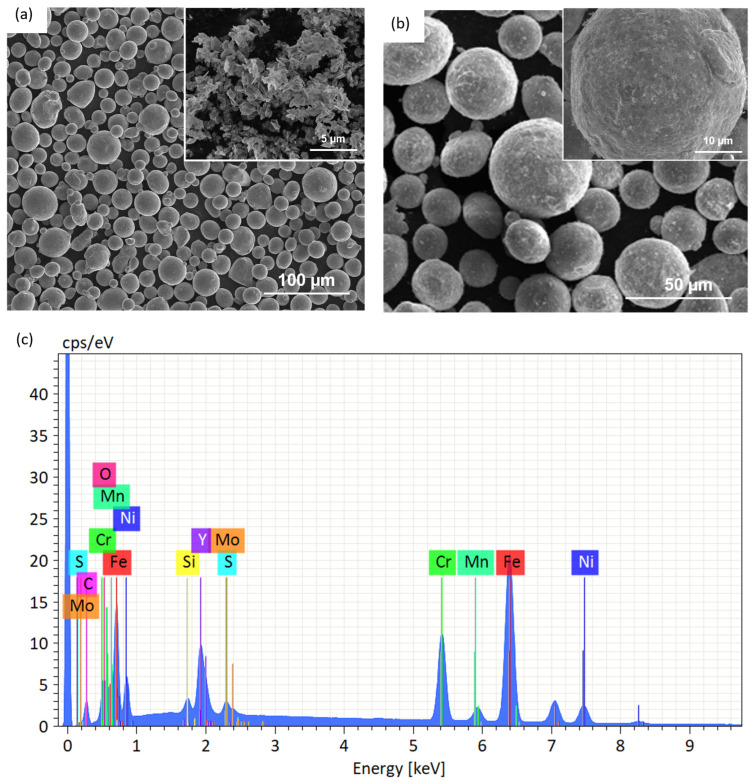
Scanning electron microscopy (SEM) images of (**a**) pre-mixing 316L and yttria nanoparticles (the inset) and (**b**) the light ball milled mixture of 316L + 0.8 wt.% of yttria powder, and the EDX spectrum (**c**) from the mixed powder after the light ball milling.

The morphologies of the pre-mixing 316L and yttria powder particles, along with the mixture of 316L and 0.8 wt.% yttria after light ball milling, are presented in Figure 1a and Figure 1b, respectively. As depicted in Figure 1a, the gas-atomized 316L powder primarily consists of spherical particles, occasionally accompanied by satellite particles, with a mean diameter of 28.9 µm. The yttria particles, shown in the inset of Figure 1a, have a prismatic shape with a mean size of 1.7 µm. After light ball milling (Figure 1b), the majority of 316L particles maintain their original spherical morphology, with only a few exhibiting deformation. The yttria particles are observed to coat the surface of the 316L particles. The inset of Figure 1b provides a higher-magnification view, revealing more detailed surface features of the lightly milled powder. The energy-dispersive X-ray (EDX) spectrum collected from the mixed powder after the light ball milling is presented in Figure 1c, which shows clear peaks for Y and O, together with the 316L SS elements.

#### 2.1.2. LPBF Process Parameter Development

The 2oneLab (2Create, Darmstadt, Germany) with a 250 W Yb: YAG fiber laser and a wavelength of 1067 nm was adopted to print eight bulk samples with the dimension of $10\times 10\times 5\;{mm}^{3}$ on a 316L SS build plate. These samples were printed using the mixed 316L + 0.8 wt.% of yttria powder and the different process parameters (laser power and scanning speed) shown in Table 2. Furthermore, the volumetric energy density (VED) corresponding to the process parameters is also listed in Table 2, which is defined by Equation (1).

(1) $$E=\frac{P}{VHT}$$

where P is the laser power, V is the scanning speed, H is the hatch spacing, and T is the layer thickness. The wide range of E is beneficial for examining the effect of the solidification time on the oxide precipitation. Other machine parameters include a spot size of 40 μm, hatch spacing of 40 μm, powder layer thickness of 25 μm, and a rotation angle of 90 degrees. The oxygen level in the LPBF chamber was consistently kept below 100 ppm by continuously purging the chamber with pure nitrogen.

#### 2.1.3. Second Phase Oxide Nanoparticle Characterization

After the LPBF process, the sample preparation for microstructural characterization was performed according to the standard metallographic procedures. The cube samples were removed from the build plate and cut in half by the wire electrical discharge machining (W-EDM) to reveal the *x*-*z* cross section of each sample (where +*x* is parallel to the surface of the build plate and +*z* is parallel to the build direction). Then, the samples were mounted in phenolic powder using the TP-7001B mounting press (Pace Technologies, Tucson, AZ, USA), and ground and polished by the NANO-2000T grinder-polisher (Pace Technologies). Furthermore, the polished samples were electroetched for 15 s by applying 15 V DC voltage, using an electrolyte solution of 10 wt.% oxalic acid. After that, a microstructural characterization of all samples was performed in an FEI Quanta 3D scanning electron microscope (SEM), manufactured by FEI Company in Hillsboro, OR, USA, coupled with energy dispersive X-ray spectroscopy (EDS). Finally, the PSD of oxide nanoparticles in the SEM images was analyzed with ImageJ (version 1.53t) [41].

### 2.2. Multiscale Modeling Framework

**Figure 2:**
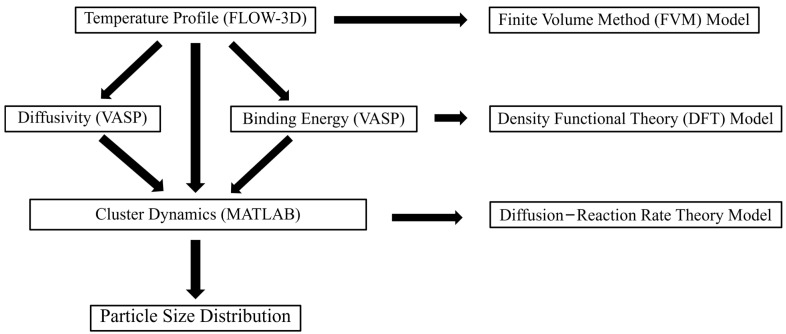
Schematic of the multiscale thermodynamic and kinetic model framework.

To understand and predict the time- and temperature-dependent oxide nanoparticle precipitation during the LPBF process, our multiscale modeling framework, illustrated in Figure 2, integrated a diffusion–reaction rate theory-based cluster dynamic (CD) model with temperature and cooling profiles. These temperature and cooling profiles, predicted for the entire melt pool, were computed using a finite volume method (FVM) model for the LPBF process. Additionally, thermodynamic and kinetic data, calculated separately through a DFT model considering molten 316L stainless steel, were incorporated. Before presenting the modeling details, it is important to note that our framework incorporated essential multiscale information and computational methods critical to understanding oxide precipitation. However, as with most modeling efforts, certain assumptions and simplifications were necessary to make the computations feasible. The goal of integrating these models is to predict the PSD of oxide nanoparticles in 316L ODS SS fabricated via LPBF.

Specifically, our modeling framework uses the following assumptions/simplifications:

Assumption (1): Only the 316L SS powder was adopted in the FVM model. Even though the 316L SS powder was coated by 0.8 wt.% of yttria powder in the experiment, the small size and weight percent of the yttria powder make it unlikely for yttria to significantly impact the temperature/cooling profile of the melt pool.

Assumption (2): The Y_2_O_3_ powder was fully melted and dissociated into monomers upon the onset of cooling. The melting point of bulk Y_2_O_3_ is 2700 K in equilibrium, and the nanoparticles usually have a lower melting point than the bulk materials. Thus, it is appropriate to assume that the Y_2_O_3_ powder is melted in most parts of the melt pool [2,3,35]. Furthermore, the Y-O nanoparticles were assumed to remain spherical during precipitation due to the strong surface-tension (interfacial energy) effect on the nanometer scale, which is indeed supported by the SEM images to be shown later.

Assumption (3): A point in the melt pool had an approximately constant cooling rate before solidification (i.e., till the melting point of 316L SS, ~1750 K), due to the short time interval between melting and solidification in the LPBF process. The cooling rate can be calculated by taking the slope between the peak temperature at that point and the melting point on the temperature versus the time profile.

Assumption (4): All Y-O nanoparticles in the liquid experienced the same Y and O concentration but a different temperature at a given instant in time. It would be adequate to assume that monomers could reach the uniform concentration in the liquid system due to the high diffusivity. Several representative temperature profiles were selected for the Y-O nanoparticle precipitation in the different regions of the melt pool, providing a more accurate prediction of the PSD than using a single temperature profile to represent the entire cooling history of the melt pool.

Assumption (5): The Y-O particles with compositions around the stoichiometric composition Y_2_O_3_ were evaluated in the CD model with a novel phase-cut method presented in Ref. [40]. Informed by experimental observation and computational work, Y_2_O_3_ had the highest thermal stability, comparing with other compositions in 316L SS [1,2,3,4,5,6,7,8,21,22,23,35,36,37,38,39,40]. Temporary and slight deviation from the Y_2_O_3_ stoichiometry was most likely occurring during the growth of the Y-O precipitates, and, hence, those close-by compositions were allowed in our cluster dynamic model.

Assumption (6): The Y-O nanoparticle precipitation was mainly controlled by the diffusion of Y and O in the molten 316L SS but not the diffusion or motion of the other elements. In contrast, during the conventional manufacturing of ODS alloys (solid-state processing), oxygen and vacancies formed bonds, with yttrium occupying the vacancies. The coarsening of nanoparticles in this process is primarily driven by yttrium diffusion along dislocations [36,37,38].

Assumption (7): There was an ample reservoir of O for the precipitation of Y-O nanoparticles during printing. Although the oxygen level was consistently kept below 100 ppm in the LPBF chamber, the residual oxygen in the chamber and the oxygen dissolved into the powder during ball milling were still affluent for forming Y-O nanoprecipitates [36].

**Figure 3:**
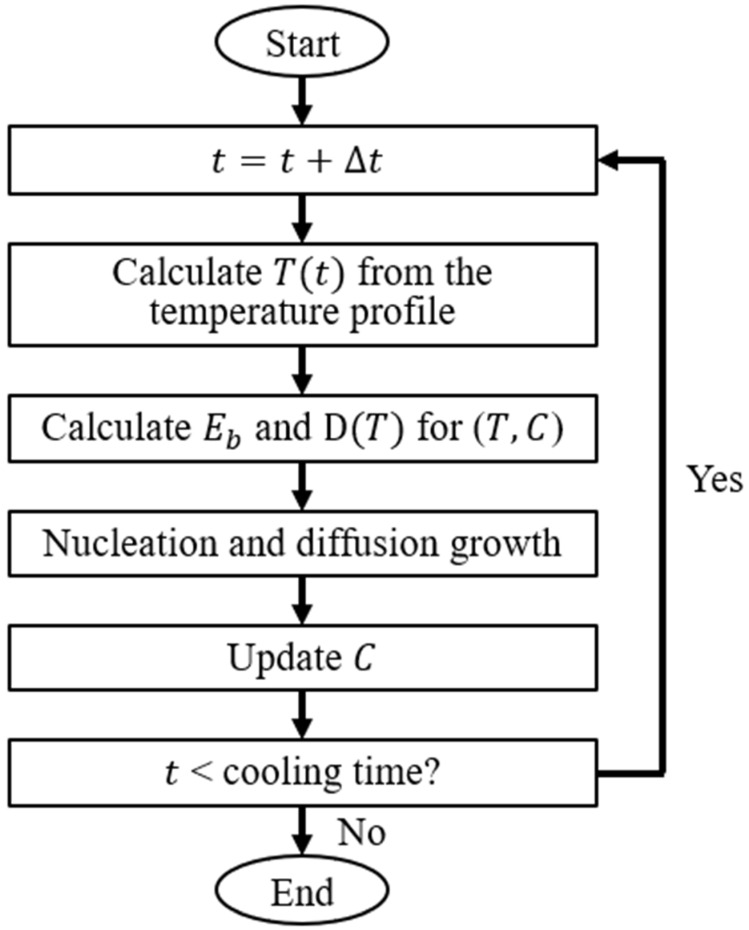
Flow chart of the cluster dynamics model to predict the oxide nanoparticle precipitation during LPBF. C is concentration, D (*T*) is temperature dependent diffusivity for Y and O monomers, and *E_b_* is the binding energy of Y-O clusters.

The flowchart of the cluster dynamics (CD) model, the part of the current multiscale modeling framework that directly predicts the concentrations of Y-O clusters of different compositions and sizes (i.e., Y-O precipitate characteristics), is given in Figure 3. Firstly, a matrix with initial concentrations of Y and O monomers and zero concentrations of non-monomer Y-O cluster compositions was created. Then, the temperature was calculated at each time step on the basis of the temperature/cooling profiles obtained from the FVM model. Later, the binding energy between the clusters and the monomers and the temperature-dependent diffusivities of the monomers were calculated based on the formulae pre-derived from the DFT and used to compute the clustering and dissociation rates of each Y-O composition. Finally, the concentrations (C) of all Y-O monomer and cluster compositions were updated by the CD model.

#### 2.2.1. Temperature Profile

The temperature/cooling profile is an important piece of information for modeling the oxide nanoparticle precipitation in additive manufacturing. Thus, researchers invested a lot of effort to measure the temperature profile in the AM process experimentally [22] and computationally [23,28,29,30,31,42,43,44]. However, it is time-consuming and technically challenging to measure the temperature profile in the experiment due to the limited size of the melt pool [22]. It is even more formidable to determine the spatially dependent temperature/cooling profiles at different points within the melt pool through the LPBF process. On the other side, in the modeling, researchers used the finite element method (FEM) and finite difference method (FDM) to calculate the temperature profiles in the AM process, simplifying the discrete powder bed as a homogenous and continuous plate on the substrate [28,29,30,31]. Some FVM models have been developed to estimate the temperature profile more accurately and comprehensively in the AM process [23,42,43,44]. Therefore, an FVM model was built in the computational fluid dynamics (CFD) software FLOW-3D (version 12.0.2.01) to simulate the single-track printing of LPBF [45]. The governing equations for mass continuity, momentum conservation, and energy conservation are given in Equations (2)–(4) [23,42,43,44].

(2) $$\nabla \cdot \left(\rho \vec{v}\right)=0$$

(3) $$\frac{\partial }{\partial t}\left(\rho \vec{v}\right)+\nabla \cdot \left(\rho \vec{v}⊗\vec{v}\right)=\nabla \cdot \left(\mu \nabla \vec{v}\right)-\nabla p+\rho \vec{g}$$

(4) $$\frac{\partial }{\partial t}\left(\rho h\right)+\nabla \cdot \left(\rho \vec{v}h\right)=q+\nabla \cdot \left(k\nabla T\right)$$

where $\vec{v}$ and $\vec{g}$ are the velocity vector and gravitational acceleration vector, respectively, ρ is the density, t is the time, μ is the viscosity, p is the pressure, k is the thermal conductivity, T is the temperature, and h is the specific enthalpy. The h can be further expressed as follows:

(5) $$h=cT+\left(1-f_{s}\right)L$$

(6) $$f_{s}=\left\{\begin{array}{c} 0\\ \frac{T_{l}-T}{T_{l}-T_{s}}\\ 1 \end{array}\right\},\;\mathrm{when}\;\begin{array}{c} T>T_{l}\\ T_{s}\leq T\leq T_{l}\\ T<T_{s} \end{array}$$

where c, L, and $f_{s}$ denote the specific heat, latent heat of melting, and fraction of the solid, respectively. Additionally, $T_{s}$ and $T_{l}$ are the solidus and liquidus temperatures, respectively.

Furthermore, the volume of fluid (VOF) is adopted to re-build the free surface at each time step, as given in Equation (7).

(7) $$\frac{\partial F}{\partial t}+\nabla \cdot (F\vec{v})=0$$

where F is the fluid fraction.

The laser source used in the experiment has a circular focal point with a ~40 μm diameter on the powder bed. Therefore, a circular laser beam with a Gaussian flux distribution was built in the current model.

(8) $$q=q_{f}exp(-{\left(\frac{r}{r_{b}}\right)}^{2})$$

where $q_{f}$ is the heat flux at the center of the laser beam, and r and $r_{b}$ denote the radial position and the inflection point, respectively.

**Figure 4:**
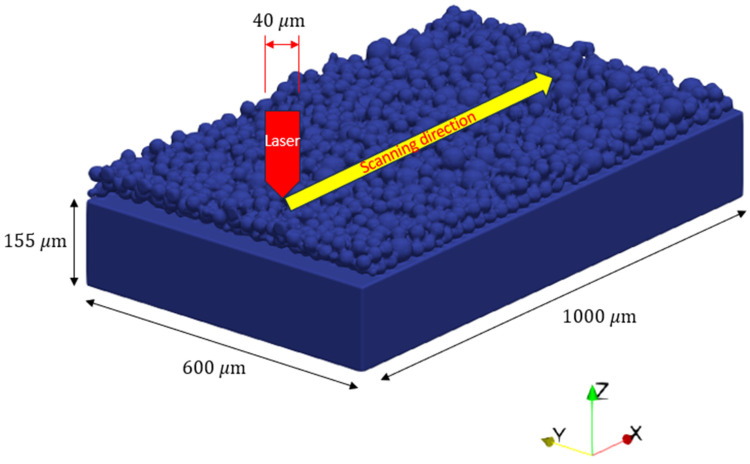
Geometry configuration of the LPBF model with a mesh size of 5 μm.

A powder bed layer of 316L representing the powders used in the experiments was constructed by the discrete element method (DEM) in the FLOW-3D DEM module and was then assembled with the 316L SS substrate as shown in Figure 4. A single track with the length of 800 μm is printed in +x direction for each simulation. The computational domain consists of 1,200,000 cells with the mesh size of 5 μm. The material properties of 316L SS (from the built-in database) used in the simulation are listed in Table 3.

#### 2.2.2. Thermodynamic and Kinetic Parameters

The precipitation and dissociation of the oxide nanoparticles were determined by the thermodynamics and kinetics in the liquid. Therefore, the binding energy of Y and O monomers to an oxide nanoparticle and the diffusivity of Y and O monomers need to be evaluated in the molten 316L SS. In this work, such calculations in the molten 316L SS were performed using the latest Vienna Ab initio Simulation Package (VASP, version 6.3.2) [46,47,48,49]. Since this study dealt with a high temperature liquid matrix, the ab initio molecular dynamics (AIMD) approach was adopted to calculate these fundamental material parameters over a wide range of disordered atomic configurations in the liquid state [4,48]. The new feature of the on-the-fly machine learning force field (MLFF) inside the VASP was employed to enhance the efficiency of AIMD in the diffusivity calculations [50].

All AIMD simulations were conducted on the high-performance computing (HPC) cluster at the College of Engineering, Oregon State University. The simulations used the NPT ensemble (constant number of atoms, pressure, and temperature), with pseudopotentials generated via the projector-augmented wave (PAW) method and the generalized gradient approximation (GGA) based on the Perdew–Burke–Ernzerhof (PBE) functional [51,52]. A plane-wave cutoff energy of 400 eV and a uniform Monkhorst–Pack k-mesh were applied. The Open Visualization Tool (OVITO, version 3.8.4) and MATLAB (version R2023b) were utilized for post-simulation 3D visualization and data analysis [53].

Given the chemical complexity of the matrix alloy (316L) and the high temperatures, it is very challenging to determine the atomic diffusivities of Y and O atoms either experimentally or computationally. Although the atomic diffusivities of Y and O in body-centered cubic (BCC) pure iron or molten pure iron have been reported in some studies [54,55,56,57,58,59], the atomic diffusivities of Y and O in the molten 316L SS have not been reported until our recent computational work [50]. The details of the DFT calculations for the atomic diffusivities of Y and O (along with Ti) in molten 316L SS can be found in Ref. [50]. A brief description of the DFT model with the MLFF for diffusivity calculations is presented here.

**Figure 5:**
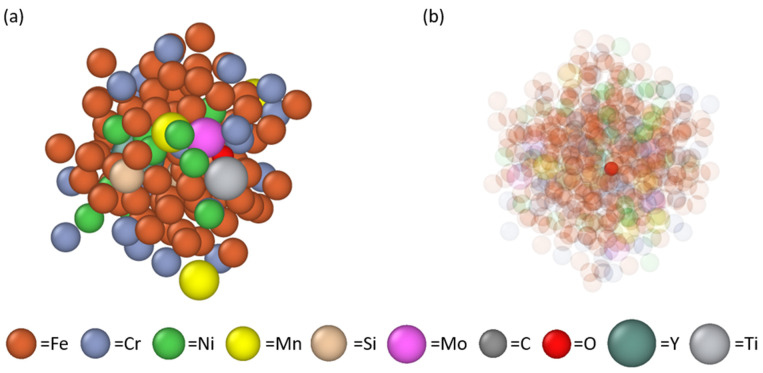
Molten state exemplary atomic configurations in the AIMD model for diffusivity calculations: (**a**) a 128 atom supercell of molten 316L SS (containing one Y, one O, and one Ti atoms) after training the MLFF, and (**b**) a 432-atom supercell of molten 316L SS (containing one O atom) after a 20 ps diffusion simulation at 2200 K.

A 4×4×4 BCC Fe supercell with 128 atoms was first created, and 42 of the Fe atoms were randomly replaced with the other constituent elements (e.g., Cr, Ni) according to the chemical composition of 316L SS as listed in Table 4 [50]. Then, one Y, one Ti, and one O atoms were introduced into this sample by randomly replacing three remaining Fe atoms (note that Ti is not directly relevant to this study but included for future work). This sample was then heated until fully melted into a disordered liquid structure (shown in Figure 5a) to train the MLFF. After that step, the MLFF was applied to the diffusion simulations in a 6×6×6 supercell (432 atoms) of 316L SS (containing one Y, one Ti or one O atom). During each diffusion simulation, the sample was held at a fixed temperature, allowing the atoms to freely migrate. Figure 5b shows an example of the disordered liquid sample containing one O atom after 20 picoseconds of diffusion. Finally, the atomic diffusivities of Y, Ti, and O in the molten 316L SS at four different temperatures were computed using the mean square displacement (MSD) method and fitted to temperature-dependent Arrhenius equations, one for each element [50].

**Figure 6:**
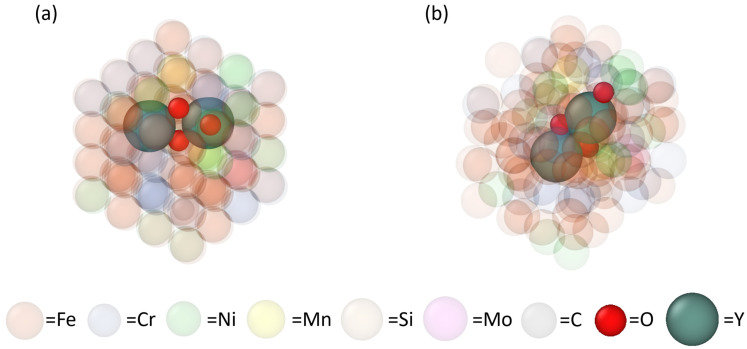
AIMD model for the total energy calculation, the 316L SS matrix with a $Y_{2}O_{3}$ embedded: (**a**) at the assembled solid state, and (**b**) after relaxation at the 2200 K liquid state.

The binding energies of Y and O to the Y-O clusters in molten 316L SS were calculated by using pure AIMD simulations only. For this calculation, we first created a 4×4×4 BCC Fe supercell with 128 atoms. Then, 15 Fe atoms at the center of the supercell were removed to make space for a bixbyite Y_6_O_9_ lattice in accordance with the structure-matched method [4]. Next, 42 atoms of the 316L constituent elements (in Table 4) were incorporated into the system by randomly replacing the remaining Fe atoms. A series of samples from $Y_{0}O_{0}$ to $Y_{6}O_{6}$ were prepared by adding different numbers of the Y and/or O atom into the bixbyite lattice. Finally, all samples were relaxed at 2200 K for 2 picoseconds with a step size of 2 femtoseconds to obtain the total energy of the equilibrium system of the molten 316L SS with a Y-O cluster embedded. Figure 6a,b exhibit the 316L SS matrix with a $Y_{2}O_{3}$ as assembled and after relaxation, respectively.

Since AIMD simulations are computationally expensive, it is challenging, and probably not necessary, to directly calculate the total energies of 316L SS samples containing every size of Y-O clusters beyond $Y_{6}O_{6}$, as this would require more than one unit cell of the bixbyite structure. Instead, the total energies we obtained for the samples with small Y-O clusters were fitted into Equation (9) for extrapolating the total energy towards bigger Y-O clusters.

(9) $$E_{total}=aY+bO+c*{\left[Y*2.47^{3}+O\right]}^{\frac{2}{3}}+d*\left(\frac{1}{2}Y^{2}-\frac{2}{3}Y\cdot O+\frac{2}{9}O^{2}\right)+e$$

where Y and O are the numbers of Y and O atoms, respectively, in the Y-O cluster. a,b,c,d, and e are fitting constants. The choice of the terms in this equation was based on considerations of volumetric and interfacial contributions of the Y and O atoms into the system energy as well as the preferred 2:3 ratio between the Y and O in forming Y-O clusters. We will show in Section 3 that this choice captured the total energy data calculated by AIMD for the small clusters very closely. After total energy extrapolation, the bind energy of one Y or O atom to a Y-O cluster was calculated by Equations (10) and (11), respectively.

(10) $$E_{bY}=E_{\left(Y-1\right)O}+E_{Y=1}-(E_{YO}+E_{SS})$$

(11) $$E_{bO}=E_{Y\left(O-1\right)}+E_{O=1}-(E_{YO}+E_{SS})$$

where $E_{b}$ is the binding energy, $E_{SS}$ is the total energy of the molten 316L SS sample with zero Y and zero O atoms. For example, the binding energy of an Y atom to a $Y_{2}O_{3}$ cluster can be calculated by $E_{Y_{1}O_{3}}+E_{Y_{1}}-(E_{Y_{2}O_{3}}+E_{SS})$, where the first three quantities are the total energies of the systems with a $Y_{1}O_{3}$ cluster, one Y atom, and a $Y_{2}O_{3}$ cluster, respectively.

#### 2.2.3. Cluster Dynamics

Precipitation phenomena are generally governed by the diffusion and interactions of the relevant species (starting from monomers) [60,61,62,63]. Here, the Y and O monomers can diffuse in the liquid 316L SS and interact with other monomers or clusters. Specifically, one monomer can combine with another monomer or a cluster to form a bigger cluster (thermodynamically stable). In the inverse way, a larger cluster can emit one monomer due to thermal dissociation. The clustering process causes the precipitation and growth of Y-O nanoparticles, and the dissociation leads to the re-dissolution of Y-O nanoparticles. Therefore, we used the following partial differential equations to describe this kinetic evolution of Y-O clusters in the liquid 316L SS [60,61,62,63].

(12) $$\frac{dC_{O=1}}{dt}=k_{O=2}^{O-}C_{O=2}+\sum_{i,j}k_{Y=i,O=j\;}^{O-}C_{Y=i,O=j}-k_{O=1}^{O+}C_{O=1}C_{O=1}-\sum_{i,j}k_{Y=i,O=j}^{O+}C_{O=1}C_{Y=i,O=j}$$

(13) $$\frac{dC_{Y=1}}{dt}=k_{Y=2}^{Y-}C_{Y=2}+\sum_{i,j}k_{Y=i,O=j\;}^{Y-}C_{Y=i,O=j}-k_{Y=1}^{Y+}C_{Y=1}C_{Y=1}-\sum_{i,j}k_{Y=i,O=j}^{Y+}C_{Y=1}C_{Y=i,O=j}$$

(14) $$\begin{array}{c} \frac{dC_{Y=1,\;\;O=1}}{dt}=0.5\;k_{O=1}^{Y+}{C_{Y=1}C}_{O=1}+0.5\;k_{Y=1}^{O+}{C_{O=1}C}_{Y=1}+k_{Y=2,O=1}^{Y-}C_{Y=2,O=1}+k_{Y=1,O=2}^{O-}C_{Y=1,O=2}-k_{Y=1,O=1}^{Y+}C_{Y=1}C_{Y=1,O=1}\\ -k_{Y=1,O=1}^{O+}C_{O=1}C_{Y=1,O=1}-{0.5\;k}_{Y=1,O=1}^{Y-}C_{Y=1,O=1}-{0.5\;k}_{Y=1,O=1}^{O-}C_{Y=1,O=1} \end{array}$$

(15) $$\begin{array}{c} \{\frac{dC_{Y=i,O=j}}{dt}=k_{Y=i-1,O=j}^{Y+}C_{Y=1}C_{Y=i-1,O=j}+k_{Y=i,O=j-1}^{O+}C_{O=1}C_{Y=i,O=j-1}+k_{Y=i+1,O=j}^{Y-}C_{Y=i+1,O=j}+k_{Y=i,O=j+1}^{O-}C_{Y=i,O=j+1}-\\ k_{Y=i,O=j}^{Y+}C_{Y=1}C_{Y=i,O=j}-k_{Y=i,O=j}^{O+}C_{O=1}C_{Y=i,O=j}-k_{Y=i,O=j}^{Y-}C_{Y=i,O=j}{-k}_{Y=i,O=j}^{O-}C_{Y=i,O=j}\} \end{array}$$

where C stands for the concentration in nm^−3^, Y and O are the numbers of Y and O atoms, respectively, and $k^{(Y\;or\;O)+}$ and $k^{\left(Y\;or\;O\right)-}$ are the clustering and dissociation rate constants for a Y-O cluster’s capturing and emitting one monomer, respectively. Note that Equation (15) is not a single equation, but rather a large system of equations for the two-dimensional composition (binary Y and O system) space defined by the (*i*, *j*) pair.

The rate constants are further determined by the following equations [39,40,60,61]:

(16) $$k_{Y=i,O=j}^{(YorO)+}=4\pi (r_{\left(YorO\right)}+r_{Y=i,O=j})(D_{\left(YorO\right)}+D_{Y=i,O=j})$$

(17) $$k_{Y=i,O=j}^{Y-}=4\pi (r_{Y}+r_{Y=i-1,O=j})(D_{Y}+D_{Y=i-1,O=j})C_{0}exp(-\frac{E_{bY}}{k_{B}T})$$

(18) $$k_{Y=i,O=j}^{O-}=4\pi (r_{O}+r_{Y=i,O=j-1})(D_{O}+D_{Y=i,O=j-1})C_{0}exp(-\frac{E_{bO}}{k_{B}T})$$

where D and $E_{b}$ are the diffusivity and binding energy, respectively, obtained from the VASP calculations, and r is the radius of the Y-O cluster. Since we consider the Y-O clusters to remain approximately spherical during precipitation, r can be calculated by Equation (19) with the radii of Y and O atoms, $r_{Y}=0.205\;nm$, and $r_{O}=0.073\;nm$ here, respectively.

(19) $$r={\left(Y*r_{Y}^{3}+O*r_{O}^{3}\right)}^{\frac{1}{3}}$$

The Y-O precipitates in LPBF typically have the size of several tens of nanometers [1,2,3,4,5,6,7,22,23,24]. To include the experimental nanoparticle size, an incredibly large composition space (from 0 to >10^9^ for both Y and O numbers) is required in the CD model, which is computationally impossible given the computing hardware available today. Therefore, all CD calculations in this study were performed up to 10^5^ Y and 1.5×10^5^ for O. After that, the peak-growth dynamics learned during each CD calculation was fitted to a mathematical function for extrapolating towards even bigger sizes of precipitates, which will be discussed in Section 3.

Even within the limits of 10^5^ Y and 1.5 × 10^5^ O, the composition space (1.5 × 10^5^ × 10^5^ points) is still too large to be solved if all the (*i*, *j*) pairs were included in the system of differential equations.

As stated in our Assumption 5 (which is supported by our energy calculations), most species in the composition space that are away from the known preferred Y:O = 2:3 ratio (corresponding to a line in the composition space) are not really going to be formed during the precipitation process. Therefore, a phase-cut method was employed to further cut out unnecessary compositions and accelerate the CD simulations significantly [60]. A full composition matrix of 12 × 18, from $Y_{1}O_{0}$ (Y monomer) and $Y_{0}O_{1}$ (O monomer) to $Y_{12}O_{18}$, was first computed and followed by a phase-cut region. In the phase-cut region, only the concentrations of 11 compositions near the preferred stoichiometry $Y_{x}O_{1.5x}$ (2:3 ratio), from $Y_{x}O_{1.5x-5}$ to $Y_{x}O_{1.5x+5}$, were calculated, and 1.5x was rounded for odd *x* values. For example, the compositions of $Y_{13}O_{15}$ to $Y_{13}O_{25}$ were incorporated in the phase-cut region for x=13. By applying the phase-cut method in the CD model, the number of compositions (that is, no. of differential equations) being solved was significantly reduced from 1.5 × 10^5^ × 10^5^ to 11 × 10^5^.

Since we assumed that all yttria powder was melted into the melt pool during heating and the oxygen was ample, the initial concentrations of $Y_{1}O_{0}$ and $Y_{0}O_{1}$ were set to be 0.333 atoms/nm^3^ and 0.666 atoms/nm^3^, respectively, according to the 0.8 wt.% of yttria powder in the experiment and the volume of the molten 316L SS sample in the VASP.

## 3. Results

### 3.1. Experimental Measurement of Particle Size Distribution

**Figure 7:**
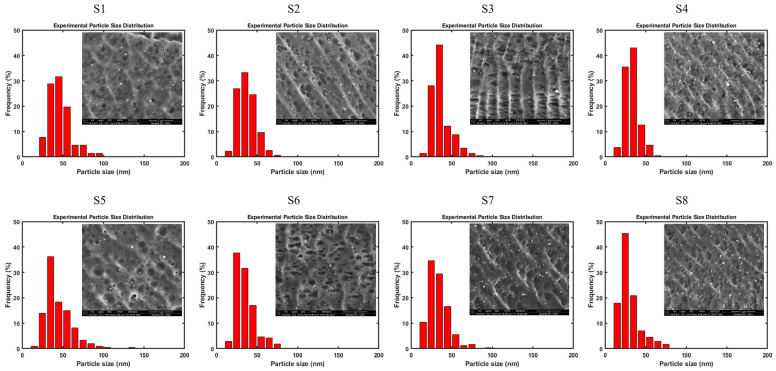
SEM micrographs and corresponding PSD histograms of Y-O nanoparticles (white spheres) in all LPBF fabricated 316L SS ODS alloys. LPBF processing parameters for S1–S8 are listed in Table 2.

To measure the PSD of Y-O precipitates in the as-printed samples, SEM micrographs were taken for all samples, as shown in Figure 7. Samples S1–S8 correspond to the LPBF processing parameters listed in Table 2. In the SEM micrographs, the Y-O precipitates are identified as numerous white spheres with approximated diameters of several tens of nanometers (10–80 nm) and uniformly distributed in the 316L SS matrix. Later, the PSD histograms were plotted by performing the statistic of the diameters of several hundred Y-O nanoparticles in the SEM micrographs, which are shown in Figure 7 as well. Due to the limitation of the SEM resolution, only the nanoparticles that are larger than 10 nm were counted. Therefore, the particle size is mostly located in the range of 10~80 nm in the PSD histogram for all samples, which is similar to other references [3,64]. Furthermore, the average diameter with the standard deviation and the total number of counted Y-O nanoparticles in each sample are given in Table 5. The data show a clear trend that the mean particle size decreases as the scanning speed increases for the same laser power.

### 3.2. Multiscale Modeling

To illustrate the predictive power of our multiscale modeling framework, one example with the laser power of 110 W and scanning speed of 300 mm/s (S2) is first discussed in detail here. The predictions for other samples are presented and validated at the end of this section.

#### 3.2.1. Temperature Profile

**Figure 8:**
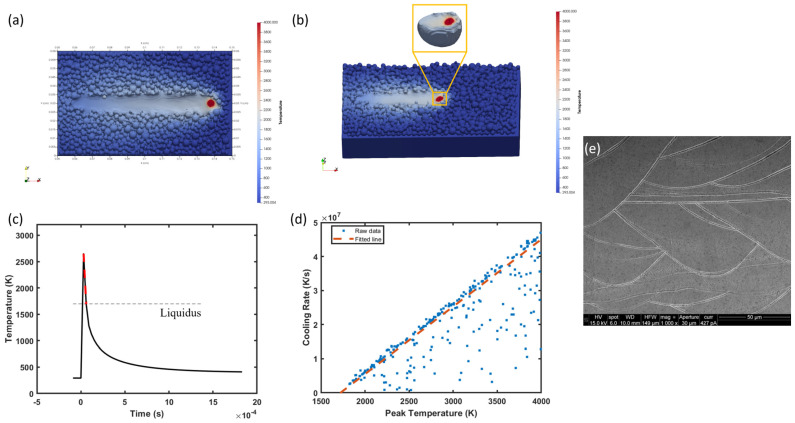
(**a**) Top view of the single track printed in FLOW-3D, (**b**) 3D view of the sample during steady printing and the isolated melt pool in the insert figure, (**c**) the temperature profile for a unit cell with a peak temperature of ~2600 K, (**d**) peak temperatures vs. cooling rates for all unit cells in the melt pool, and (**e**) an SEM micrograph of the cross section of the S2 sample with multiple tracks.

A length of 800 μm track is printed with 110 W laser power and 300 mm/s in FLOW-3D, as shown in Figure 8a. The width of this track is ~120 μm, which is close to the experimental width (~140 μm), averaged from 13 tracks, as shown in Figure 8e. It is typical that there is an extremely hot area (>4000 K) under the laser beam in the modeling due to the heat concentration at the center of Gaussian heat flux. This area is excluded from the temperature profiles later. To capture the temperature profiles in the melt pool, we isolate the melt pool during the middle stage of the LPBF process and export the temperature profiles that are experienced by all unit cells in the melt pool, as illustrated in Figure 8b. After that, the cooling rate in the temperature profile is calculated by taking the slope between the peak temperature and the melting point (~1750 K) of 316L SS for all unit cells. For instance, the temperature profile in Figure 8c is plotted for a unit cell experiencing a peak temperature of ~2600 K. The slope of the red dash line provides a good estimate for the cooling rate at this point (represented by this unit cell). The blue squares in Figure 8d show a nearly linear relationship between the peak temperatures and cooling rates at different points within the melt pool, that is, a point would have a higher cooling rate if it experienced a higher peak temperature in the melt pool. Therefore, the peak temperatures and cooling rates are fitted into a function (Equation (20)) by linear regression, as marked by the red dash line in Figure 8d.

(20) $$CR=1.98\times 10^{4}\times T_{p}-3.40\times 10^{7}$$

where CR and $T_{p}$ are the cooling rate and peak temperature, respectively, experienced by any point in the melt pool. As will be discussed later, some representative peak temperatures, representing different positions in the melt pool, and their corresponding cooling rates will be used in the CD model.

#### 3.2.2. Diffusivity and Binding Energy

As mentioned in Section 2.2.2, the diffusivities of Y and O monomers in the molten 316L SS were calculated by AIMD with the latest feature MLFF in the VASP and published in our previous work [50]. The temperature-dependent diffusivities of Y and O in the liquid 316L SS are given in Equations (21) and (22), respectively.

(21) $$D_{Y}(T)=4.40\times 10^{-4}exp(-\frac{4.62\times 10^{4}}{RT})$$

(22) $$D_{O}(T)=2.78\times 10^{-2}exp(-\frac{1.06\times 10^{5}}{RT})$$

where R is the universal gas constant, and T is the absolute temperature. Thus, the diffusivities of Y and O monomers can be calculated on the fly for any time instant (temperature known from Flow-3D predictions) during a CD modeling process.

For a binding energy calculation, the total energies of the systems containing Y-O clusters from $Y_{0}O_{0}$ to $Y_{6}O_{6}$ were computed by conducting pure AIMD simulations, as explained in Section 2.2.2. More specifically, the total energies of the systems with Y-O clusters were computed by taking the average total energy from the last 500 fs of the relaxation process to ensure a stabilized melt pool. The values are listed in Table 6. $Y_{0}O_{0}$ represents the 316L SS matrix without any Y-O clusters.

**Figure 9:**
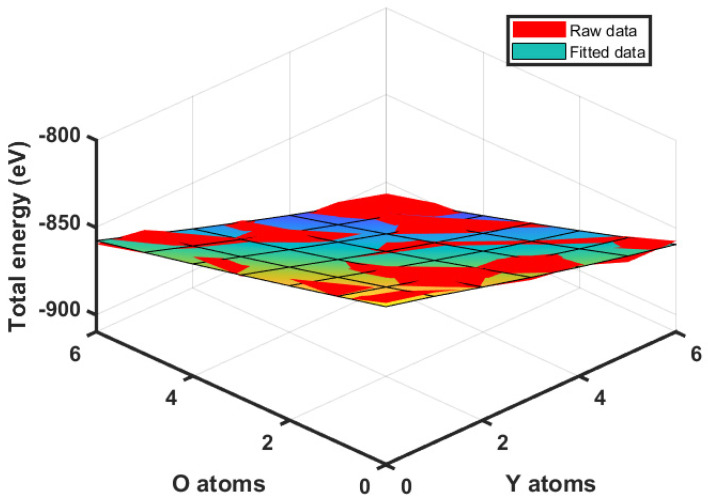
Total energies of the systems containing Y−O clusters with various numbers of Y and O atoms, calculated by pure AIMD in the VASP (red surface) and the fitted equation (multi-colored surface).

Due to the high computational cost of pure AIMD simulations, it is challenging (and most likely unnecessary) to calculate the total energy of the system with a cluster that is larger than $Y_{6}O_{6}$ in the 316L SS matrix. Therefore, the total energies in Table 6 are fitted into the mathematical expression in Equation (9), for extrapolating the total energy to systems containing larger Y-O clusters. The fitted expression is given by Equation (23). The total energies in Table 6 directly calculated by AIMD and those re-calculated by the fitted Equation (23) are plotted together in Figure 9 as the red and multi-colored surfaces, respectively. With the total energy determined, the binding energy of one Y or O atom with an Y-O cluster is then calculated by Equations (10) and (11) in the CD model.

(23) $$E_{total}=-9.424Y-7.218O+0.4*{\left[Y*2.47^{3}+O\right]}^{\frac{2}{3}}+0.4552*\left(\frac{1}{2}Y^{2}-\frac{2}{3}Y\cdot O+\frac{2}{9}O^{2}\right)-819.2$$

#### 3.2.3. Cluster Dynamics Predictions

The temperature profiles computed in FLOW-3D and the diffusivity and binding energy expressions derived from ab initio simulations are finally incorporated into the CD model to predict the precipitation of the Y-O nanoparticles during the cooling process.

**Figure 10:**
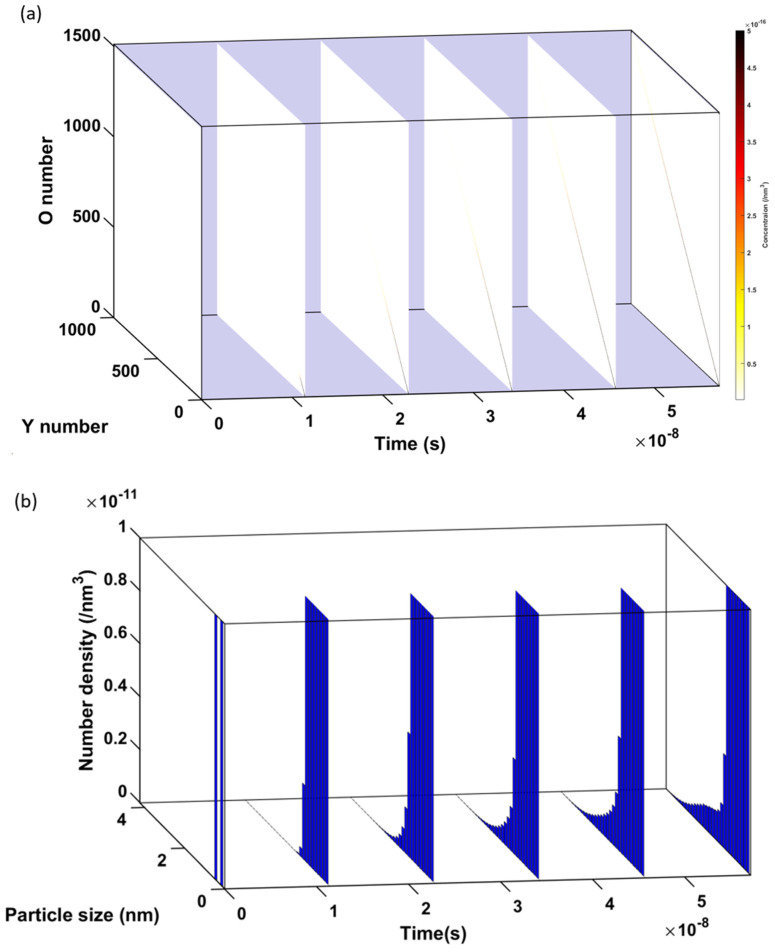
(**a**) Cluster evolution in a 1000 × 1500 Y−O composition matrix during the first 1 K drop, and (**b**) corresponding PSD histograms of Y-O clusters.

As stated in Section 2.2.3, to increase the computational efficiency, the phase-cut method is employed to skip most of the unnecessary compositions that are away from the Y:O = 2:3 ratio (hence unfavored by thermodynamics) while keeping those that are close enough to the 2:3 ratio (11 allowed O numbers for each Y number). The phase-cut method is demonstrated in Figure 10a using a superficially small system size (1000 Y × 1500 O), which shows the very narrow composition ‘line’ (black in the color bar) in the phase space (six white standing planes) within the first 1 K temperature-drop (2600 K to 2599 K, a step size of 0.2 K). The growth of the line indicates the Y-O cluster evolution controlled by the clustering and competing dissociation during the cooling process. The width of the growth line remains very narrow, in close proximity to the preferred Y2:O3 stoichiometry, justifying the omission of most compositions in the white background of the phase space via the phase-cut method. The cluster evolution is also revealed by the corresponding PSD histograms in Figure 10b (where a small concentration scale is intentionally chosen to show the formation of a nucleation peak by the last timestep included). At time zero, those two bins represent the initial concentrations of Y and O monomers. As Y-O clusters are formed by the diffusion and interactions of the monomers, each size bin contains a range of Y-O clusters with different compositions (Y and O numbers).

In our actual CD calculations, instead of 1000 Y × 1500 O, the maximum number of Y in the composition space considered is 10^5^, and, correspondingly, the maximum number of O is 1.5 × 10^5^. This calculation corresponds to a maximum cluster size of ~19 nm, which is still on the lower side of the 10–80 nm particle size range observed in experiments. We will discuss how to extend the CD predictions to the full experimental size range later.

Since different points (unit cells in Flow-3D) within the melt pool experience different peak temperatures and cooling rates, we choose multiple points, namely, those experiencing a peak temperature of 2400 K, 2600 K, 2800 K, 3000 K, 3200 K, and 3500 K, to perform separate CD modeling. And the CD predictions for these individual points will be finally combined by applying volume fractions of points (unit cells) with similar thermal histories to these representative points as the weighting factors, which will be discussed later.

**Figure 11:**
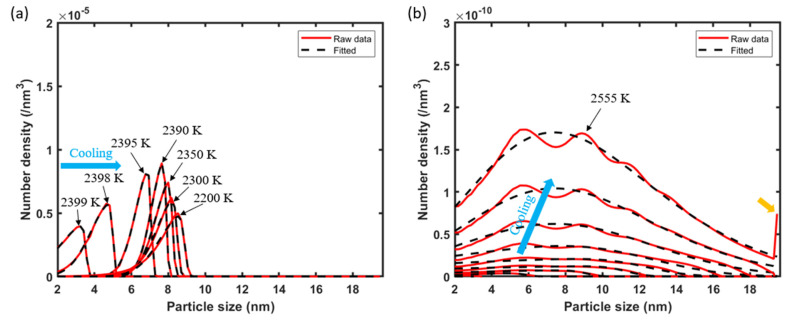
Y−O cluster evolution during solidification (**a**) from 2400 K to 2200 K (plotted for marked temperatures), and (**b**) from 2600 K to 2555 K (plotted every 5 K drops in temperature). The blue arrows indicate the nanoparticle evolution during the cooling process, and the yellow arrow indicates that the nanoparticle evolution has reached the composition boundary.

Figure 11a and Figure 11b present the PSD curves directly predicted by the CD modeling in the early stage of cooling for the unit cells that have experienced peak temperatures of 2400 K and 2600 K, respectively, according to Flow-3D. In the CD modeling, the diffusivities of the Y and O monomers and the capturing and emission rates of Y-O clusters are updated every 5 K drops in temperature using their temperature dependences established earlier. In Figure 11a, small Y-O clusters rapidly form and reach a relatively high concentration after the first 10 K cooling due to the high thermodynamic driving force at 2400 K. This high concentration of early formed clusters reduces the monomer concentrations significantly and slows down the growth upon further cooling (e.g., 2390 K to 2200 K in the figure). In contrast, the Y-O clusters in Figure 11b grow quickly at much lower concentrations, due to the faster kinetics (higher monomer diffusivities) and lower thermodynamic driving force at 2600 K. One detail to note here is that, in Figure 11b, the CD predicted cluster evolution has reached the boundary of the allowed composition space at 2555 K, as evidenced by the increased number density near the maximum size (which is an artifact due to the prescribed composition space). In this case, CD modeling is stopped at 2555 K, and only the predictions prior to the boundary accumulation occurred are used for further processing.

To extend the CD predictions beyond the early cooling stage and the 19 nm maximum size set by the employed composition space, the PSD curves at different timesteps (i.e., temperatures) from the CD calculations for one point in the melt pool are each fitted into a skewed Gaussian distribution (Equation (24)).

(24) $$C\left(x\right)=\frac{2}{d*\sqrt{2*\pi }}*\exp\left(-\frac{{\left(\frac{x-\xi }{\omega }\right)}^{2}}{2}\right)*\frac{1}{2}*\left[1+erf(\alpha *\frac{x-\xi }{\omega *\sqrt{2}})\right]$$

where $C\left(x\right)$ is the concentration of the nanoparticle with a diameter of x. d, ξ, ω, and α are fitting parameters. The clusters that are smaller than 2 nm (including Y and O monomers) are excluded from this calculation as they are too small to relate to experiments. The series of values for each parameter at different times are further fitted into time-dependent functions d(t), ξ(t), ω(t), and α(t) to form a time-dependent Equation (25). The PSD re-calculated by Equation (25) for the two representative points (with a peak temperature of 2400 K and 2600 K) discussed above at the beginning of the cooling process is included in Figure 11 as the black dashed line. These fittings show good ability to capture the PSD shape and position for all the timesteps.

(25) $$C\left(x,t\right)=\frac{2}{d(t)*\sqrt{2*\pi }}*\exp\left(-\frac{{\left(\frac{x-\xi (t)}{\omega (t)}\right)}^{2}}{2}\right)*\frac{1}{2}*\left[1+erf(\alpha (t)*\frac{x-\xi (t)}{\omega (t)*\sqrt{2}})\right]$$

**Figure 12:**
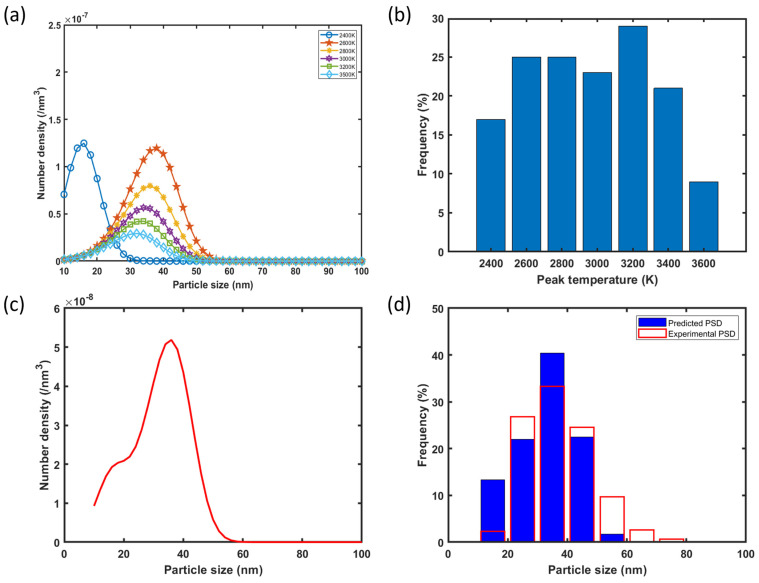
(**a**) Predicted PSD curves for different representative points (peak temperatures) in the melt pool of sample S2 after solidification, (**b**) statistics of peak temperatures in the melt pool, (**c**) the combined PSD, and (**d**) the comparison of the predicted and experimentally measured PSD for sample S2.

With Equation (23), the final PSD of Y-O nanoparticles for each representative point in the melt pool after solidification is then calculated using the time required for that point to reach the melting point of 316L SS, which is known from the thermal history predicted by Flow-3D. The obtained final PSDs for the above mentioned six representative points in the melt pool of Sample S2 are shown in Figure 12a. As mentioned before, the PSD for the point with the peak temperature of 2400 K is smaller than the others due to the lower growth rate. The very high peak temperature points (e.g., 3200 K, 3500 K) experience higher cooling rates, thus a shorter precipitation time (note that the precipitation of Y-O clusters cannot start above the melting point of Y_2_O_3_, 2700 K). Therefore, those points exhibit lower number densities and sizes of the nanoparticles compared with the points experiencing the intermediate peak temperatures.

To combine the PSDs for the individual representative points into one for the entire melt pool, the volume fractions corresponding to the representative points are determined by counting the number of unit cells in Flow-3D with similar peak temperatures and calculating their frequency, as shown in Figure 12b. After that, the final PSD in as-fabricated S2 is computed by combining the PSD curves in Figure 12a with the volume fractions as the weighting factors. It is important to note that nanoparticles smaller than 10 nm are excluded from the predicted PSD curve since those nanoparticles are not detectable in SEM. As shown in Figure 12d, the agreement between the predicted and the experimentally measured PSD is fairly good, especially in the most-probable size intervals, 20–30, 30–40, and 40–50 nm. Based on the predicted PSD, the mean particle size is predicted to be 32 nm, which is fairly close to the experimentally determined mean size of 37 nm.

The PSDs for S1–S8 were all calculated by the current model following the above described steps, and the predicted and experimentally determined mean particle diameters are listed in Table 7. The predictions overall show the same trend of decreasing particle size with increasing scanning speed (under a fixed laser power) as observed in experiments. With the laser power fixed, the higher scanning speed leads to a bigger volume fraction of unit cells in the melt pool that experience lower peak temperatures (e.g., 2400 K), which further result in smaller Y-O nanoparticles due to a stronger thermodynamic driving force (as well as slower kinetics), as discussed earlier. However, smaller nanoparticles are observed while increasing the laser power and keeping the VED constant, like S1 and S6, because the higher laser power increases the melt pool size [64]. The larger melt pool possesses better cooling efficiency and higher cooling rates, and, thus, shorter precipitation times and smaller nanoparticles. As shown in Table 7, the predicted mean diameters are basically within ±20% of the experimental values for all the eight different samples (i.e., printing conditions), which is considered good agreement given the complexity of the physics involved and the statistical uncertainty in identifying the nanoparticles and measuring their individual sizes from SEM images. This finding demonstrates a good predictive power of the present multiscale modeling framework.

## 4. Discussion

**Figure 13:**
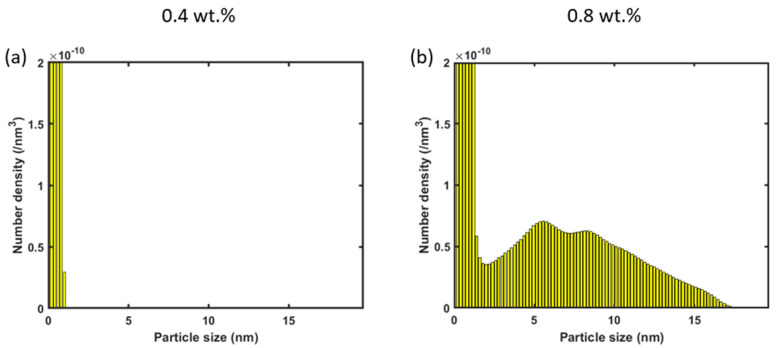
PSD (histograms) after CD simulations from 2600 K to 2570 K with initial concentrations of (**a**) 0.4 wt.% and (**b**) 0.8 wt.% of yttria powder.

It was assumed that all the yttria (0.8 wt.%) initially mixed with the 316L SS powder was captured in the melt pool, and that was used to set the initial concentration of Y monomers in the CD model. However, some amount of yttria powder may get lost during the experimental processes, such as (light) ball milling and sputtering during printing [2,22,27]. The driving force of the Y-O clustering is lowered by the smaller initial concentration in the CD model. To illustrate the effect of this factor on the PSD, a CD simulation with 0.4 wt.% of yttria powder was conducted on the basis of the temperature profile of a unit cell in the melt pool experiencing a peak temperature of 2600 K. As shown in Figure 13a, with 0.4 wt.% of yttria, only small Y-O clusters (<1 nm) form by the time the temperature drops to 2570 K. In contrast, with 0.8 wt.% of yttria, much larger clusters are already formed during the first 30 K cooling, as shown in Figure 13b. This finding implies that it is important, for the purpose of accurately predicting nano-oxide evolution for an arbitrary printing condition, to investigate exactly how much yttria powder is lost in experiments, even though it may be challenging.

Another assumption made in this study is that the thermal history of points (unit cells) inside the melt pool is not affected by the addition of the 0.8 wt.% of yttria powder [65]. Indeed, our Flow-3D calculations of the thermal history were based on 316L SS only. While the assumption is reasonable considering the small fraction of the yttria powder, it may warrant future effort to investigate the effect of the yttria powder on the thermal history of the melt pool by explicitly including both the 316L SS and the yttria powders in the Flow-3D modeling.

In general, the mechanical and creep properties of ODS alloys are enhanced, especially at elevated temperature, by a smaller size and higher number density of oxide nanoparticles. The number density of the oxide particles was not determined in experiments here due to technical limitations. However, the number density of ~10^−8^–10^−7^/nm^3^ predicted by our multiscale modeling framework is on the same order as the experimental measurements in other reports [22,66,67].

## 5. Conclusions

To summarize, a physics-based multiscale modeling framework was developed to investigate the precipitation of Y-O nano-oxides in molten 316L stainless steel (SS) and predict the particle size distribution (PSD) of the oxide nanoparticles in as-fabricated 316L SS ODS alloys. Specifically, a cluster dynamics (CD) model is used to simulate the simultaneous clustering and re-dissolution of Y-O particles and their net evolution during the cooling of the melt pool. This model incorporates thermal history data from a finite volume method (FVM) model and thermodynamic and kinetic parameters from density functional theory (DFT) models. The key points from this work are summarized below:

The FVM model in FLOW-3D was used to compute the temperature field and cooling history of the entire melt pool, providing crucial input to the CD model. This approach reduces the experimental workload typically required to measure temperature fields.

Measuring temperature-dependent thermodynamic and kinetic parameters, such as solute atomic diffusivities and cluster binding energies in multicomponent alloy systems at high temperatures, is challenging. These parameters for Y and O in molten 316L SS were computed through DFT calculations. For larger Y-O clusters, the high computational cost of the DFT was mitigated by extrapolating data from smaller clusters.

The CD model offers valuable insight into the continuous evolution of Y-O clusters, accounting for both clustering and re-dissolution processes. To manage the computational complexity of this detailed model, a phase-cut method was applied, reducing unnecessary compositions from the composition space. Despite this action, direct CD modeling remains limited to particle sizes of ~19 nm and the early stages of the cooling process (~50 K drop in temperature). A novel extension method, utilizing a time-dependent skewed Gaussian equation, was developed to predict the evolution of larger particle sizes throughout the entire cooling process.

The predicted mean particle sizes for eight different LPBF conditions are within ±20% of the experimental results, which is fairly good considering the statistical uncertainty in resolving and analyzing the dispersed particles in experiments. The predicted histograms (PSDs) also align well with experimental data, particularly within the most probable particle size range of 20–50 nm. Furthermore, the number density of ~10^−8^–10^−7^/nm^3^ predicted by our multiscale modeling framework aligns well with the order of magnitude observed in experimental measurements reported in other studies. The overall agreement with experiments highlights the reliability of our multiscale modeling framework and its predictive power suitable for guiding future experiments to achieve targeted particle characteristics.

This multiscale modeling framework could be extended to the Laser Directed Energy Deposition (LDED) process by incorporating an LDED model within FLOW-3D. Additionally, the current cluster dynamics (CD) model, developed for the Y-O binary system, can also be extended to a ternary oxide system, such as Y-Ti-O, by either expanding the compositional space from two dimensions to three dimensions or simplifying the ternary system to a pseudo-binary system (Y-Ti) based on the significantly higher diffusivity of O in molten 316L SS [34,50] and, correspondingly, the lesser role in controlling the overall precipitation kinetics than Y and Ti.

## Figures and Tables

**Table 1:** Chemical composition of 316L stainless steel powder (wt.%).

Element	Cr	Ni	Mo	Si	Mn	O	C	S	Fe
wt.%	17.05	12.50	2.40	0.69	0.86	0.02	0.016	0.03	Bal.

**Table 2:** Experimental laser power and scanning speed combinations used in the LPBF process and the corresponding VED values.

Sample (S) No.	1	2	3	4	5	6	7	8
Laser Power (W)	110	110	110	110	220	220	220	220
Scanning speed (mm/s)	150	300	600	900	150	300	600	900
VED (J/mm^3^)	733.3	366.7	183.3	122.2	1467	733.3	366.7	244.4

**Table 3:** Material, thermal, and physical properties of 316L SS [from FLOW-3D database].

Property	Unit	Value
Density	kg/m^3^	8119.6 − 0.511 T, T < 1697.15 K
		8212.6 − 0.773 T, T > 1697.15 K
Heat capacity	J/(kg·K)	430.67 + 0.1785 T, T < 1697.15 K
		830, T > 1697.15 K
Thermal conductivity	W/(m·K)	11.44 + 0.0136 T, T < 1697.15 K
		30.5, T > 1697.15 K
Viscosity	Pa·S	8 × 10^−3^
Solidus temperature	K	1674.15
Liquidus temperature	K	1697.15
Latent heat of melting	J/kg	2.6 × 10^5^
Surface tension	N/m	1.87
Thermocapillary coefficient	N/(m·K)	−4.5 × 10^−4^
Laser absorption rate		0.35, T < 1523 K
		0.4, T > 1608 K

**Table 4:** Chemical composition of 316L SS and the number of atoms for the constituent elements in the 4×4×4 supercell.

Elements	Fe	Cr	Ni	Mn	Si	Mo	C	Total
Nominal composition (at.%)	Bal	17.90	9.33	1.99	1.46	1.15	0.36	100
No. of atoms	86	23	12	3	2	1	1	128

**Table 5:** Average diameter (with standard deviation) and number of counted Y-O nanoparticles shown in Figure 7.

Sample	Average Diameter (nm)	No. of Nanoparticles
S1	46 ± 14	218
S2	37 ± 11	309
S3	37 ± 12	206
S4	33 ± 9	214
S5	44 ± 17	207
S6	35 ± 12	212
S7	33 ± 12	306
S8	30 ± 13	245

**Table 6:** Total energies (eV) of the systems with Y-O clusters.

	Y	0	1	2	3	4	5	6
O								
0		−817.048	−823.657	−833.402	−841.910	−846.223	−856.936	−857.579
1		−826.397	−834.067	−839.231	−847.445	−853.352	−863.636	−867.696
2		−828.557	−842.885	−846.728	−859.006	−865.914	−870.038	−878.215
3		−839.811	−850.337	−855.929	−863.466	−870.766	−880.074	−885.019
4		−841.357	−855.324	−866.300	−871.434	−879.707	−882.049	−896.388
5		−852.025	−860.451	−868.924	−876.428	−893.683	−892.432	−899.229
6		−859.469	−864.055	−878.310	−884.000	−896.340	−898.704	−906.546

**Table 7:** Predicted and experimental mean diameters of Y-O nanoparticles in as-printed LPBF ODS 316L SS.

	Predicted Mean Diameter (nm)	Experimental Mean Diameter (nm)	Error (%)
S1	41	46	−10.87
S2	32	37	−13.51
S3	32	37	−13.51
S4	26	33	−21.21
S5	37	44	−15.91
S6	37	35	5.71
S7	37	33	12.12
S8	36	30	20.00

## Data Availability

The original contributions presented in the study are included in the article, further inquiries can be directed to the corresponding author.
